# Examining bidirectional associations between perceived social support and psychological symptoms in the context of stressful event exposure: a prospective, longitudinal study

**DOI:** 10.1186/s12888-022-04386-0

**Published:** 2022-11-28

**Authors:** Sarah Thomas, Philipp Kanske, Judith Schäfer, Katrin Veronika Hummel, Sebastian Trautmann

**Affiliations:** 1 Institute of Clinical Psychology and Psychotherapy, Faculty of Psychology, Technische Universität Dresden, Dresden, Germany; 2 Max Planck Institute for Human Cognitive and Brain Sciences, Leipzig, Germany; 3 Department of Psychology, Faculty of Human Science, Medical School Hamburg, Am Kaiserkai 1, 20457 Hamburg, Germany; 4 ICPP Institute of Clinical Psychology and Psychotherapy, Medical School Hamburg, Hamburg, Germany

**Keywords:** Social support, Stress, psychological, Occupational stress, Stress disorders, post-traumatic, Depression, Anxiety, Longitudinal studies, prospective studies, military personnel, Military deployment

## Abstract

**Background:**

After stressful event exposure, higher perceived social support is a well-established correlate of decreased risk for psychological symptoms, including depressive, anxiety and posttraumatic stress (PTS) symptoms. However, longitudinal data on the direction of this association and the stability of perceived social support are scarce and have yielded mixed results, with a particular lack of prospective studies. We aimed to investigate changes in perceived social support and bidirectional associations between perceived social support and psychological symptoms in a prospective, longitudinal study.

**Methods:**

A sample of German soldiers was assessed before and after deployment to Afghanistan. Group-based trajectory modelling was used to investigate the stability of perceived social support and to identify possible distinguishable trajectories of perceived social support. Bidirectional associations between perceived social support (general and workplace) and psychological symptoms (depressive, anxiety and PTS) were examined using gamma regressions.

**Results:**

Average levels of perceived general social support did not change, while perceived workplace social support increased slightly (t(344) = 5.51, *p* < .001). There were no distinguishable trajectories of perceived social support. Higher perceived general (Mean ratio (*MR)* = 0.84, 95% CI = [0.74, 0.95]) and workplace social support (*MR* = 0.82, 95% CI = [0.72, 0.92]) predicted lower depressive symptoms, but not anxiety or PTS symptoms. Only higher PTS (*MR* = 0.95, 95% CI = [0.91, 0.99]) and higher depressive symptoms (*MR* = 0.96, 95% CI = [0.93, 0.99]) predicted lower perceived general social support.

**Conclusions:**

Perceived social support can remain relatively stable under exposure to environmental stressors such as military deployment. Higher perceived social support could protect against depressive symptoms via a stress-buffering mechanism, while support may need to be more tailored to individual needs for a protection against PTS symptoms. Individuals with elevated depressive and PTS symptoms might have impaired abilities or opportunities to access social support after stressful event exposure. Future studies could investigate distressing social emotions and associated maladaptive social cognitions as possible mechanisms in the association between symptoms and lower perceived social support. Especially with respect to PTS symptoms, future studies could focus on conditions that enable individuals to benefit from social support.

**Supplementary Information:**

The online version contains supplementary material available at 10.1186/s12888-022-04386-0.

## Background

Exposure to severe stressful events is a well-established risk factor for the development and maintenance of different psychological symptoms, including, depressive, anxiety and posttraumatic stress (PTS) symptoms [1–7]. However, most individuals manage to adjust well to the exposure of even severe stressful events [8, 9]. In this context, perceived social support is discussed as one of the most important resilience factors, particularly with respect to depressive and PTS symptoms [10–13]. Perceived social support captures individuals’ general beliefs about the availability of support and their satisfaction with that support [14]. It could function as a buffer against the possible adverse effects of stressful event exposure by strengthening an individual’s perceived resources and perceived ability to cope with the event [15]. However, this view has been mainly supported by the cross-sectional association between higher perceived social support and lower psychological symptomatology after stressful event exposure [14]. Longitudinally, both the stability of perceived social support itself as well as its interplay with psychological symptoms in the context of stressful event exposure are still not well-understood.

Some authors have discussed perceived social support as being a relatively stable trait [16, 17] that shares commonalities with personality dispositions [18]. However, perceived social support can also be seen as variable over time, largely depending on living circumstances, individuals’ current mental health and exposure to positive or negative life events [19–21]. In line with this, severe stressful event exposure could have an important impact on perceived social support [22]. Dealing with such events can be a potential burden and challenge for a victim’s social network and, in addition, individuals coping with severe stressful event exposure might have difficulties to engage in trusting relationships [19, 21]. However, there are heterogeneous study results on whether perceived social support decreases, increases or remains stable in the context of stressful event exposure [19, 21, 23, 24]. Diverging findings could indicate that there are different social support trajectories including stable and variable courses. However, due to a lack of prospective studies, the extent to which perceived social support remains stable in the context of stressful event exposure, is still not well-known.

Regarding the longitudinal association between perceived social support and psychological symptoms, different assumptions exist as well, especially with respect to the question of directionality. Most studies assume a direction from higher perceived social support to lower psychological symptoms after stressful event exposure [14, 25]. However, there is also evidence for the opposite direction, that is from higher psychological symptoms to lower perceived social support [21, 25, 26]. Psychological symptoms, such as PTS and depressive symptoms, can affect social behaviour and cognition and might therefore also negatively influence perceptions of social support [27, 28]. Beyond the question of directionality, some authors suggest that the longitudinal association between perceived social support and psychological symptoms, after stressful event exposure, is rather attributable to common causes of both perceived social support and psychological symptomatology than to causal processes [24]. A likely candidate to influence both psychological symptoms and perceived social support is personality [24]. Among personality variables, particularly neuroticism could be a common cause of both perceived social support and psychological symptoms [18, 24, 29] as it predicts higher psychological symptoms after stressful event exposure [30] and is also related to lower perceived social support [18]. Another possible common cause could be past exposure to extremely stressful and traumatic events. Higher exposure to traumatic events is known to contribute to psychopathology after subsequent stress exposure [31] and is also associated with lower perceived social support [19]. So far, only few studies have investigated bidirectional associations between perceived social support and psychological symptoms in the context of stressful event exposure and have revealed mixed results. Some studies found that psychological symptoms predict perceived social support, but not vice versa [32–34], others demonstrated the contrary [35, 36], some studies found evidence for bidirectional associations [21, 37, 38] and others found evidence for the association between perceived social support and psychological symptoms being rather due to stable individual differences than to within-person changes [24, 39].

Taken together, perceived social support is a well-established correlate of decreased risk for psychological symptoms after stressful event exposure. However, there is a lack of prospective, longitudinal studies. Consequently, both the stability of perceived social support as well as its interplay with psychological symptoms over the course of stressful event exposure are still debated.

Therefore, we aimed to investigate changes in perceived social support as well as bidirectional associations between perceived social support and PTS, depressive and anxiety symptoms in the context of stressful event exposure in a prospective, longitudinal study. The study was conducted in a sample of German soldiers before and after deployment to Afghanistan. Specifically, the aim was to investigate 1) whether, there is a change in average levels of perceived social support in the course of deployment, 2) whether there are distinguishable subgroups of individuals with different trajectories of perceived social support, 3) whether there is a longitudinal association between perceived social support before and psychological symptoms following deployment and vice versa, and 4) whether longitudinal associations are still present when adjusting for neuroticism and previous traumatic events as possible common causes of perceived social support and psychological symptoms.

## Methods

### Participants

We analyzed data originally collected as part of a previous prospective-longitudinal component of a German study program investigating mental health consequences of military deployment in German soldiers [40]. As a vast majority of soldiers are exposed to at least one stressful event during deployment [41] the sample is well-suited to study predictors and consequences of mental health conditions related to stress exposure. A comprehensive description of the entire study design can be found elsewhere [40]. Soldiers had to be at least 18 years old to be included in the study program. Since the low proportion of females in the German military would not have permitted adequate subgroup analysis, only male soldiers were included. A stratified random sample of 895 soldiers was drawn from a total of 4200 soldiers of the 26th and 27th contingents of the German ISAF mission in Afghanistan in 2011/2012. Combat units, representing a high-risk group for adverse psychological consequences of deployment, were sampled with a greater probability to ensure sufficiently high rates of diagnosable mental health problems in the analysis sample [40, 41].

To be eligible for the study, soldiers had to be present at their home base locations during the assessment periods. Furthermore, for financial and logistical reasons, only locations with a sufficient number of soldiers (*n* > 50) could be included. Of the 895 soldiers, 117 soldiers were ineligible because they were not stationed in one of the nine target locations (2.6%), were on sick leave (7.7%), on holiday (23.1%) or at training courses (34.2%). This resulted in 778 eligible soldiers of whom *n* = 124 refused to participate, *n* = 33 did not appear at the time their assessment was scheduled, and *n* = 3 were excluded due to female gender. Thus, 618 soldiers finally participated in the baseline assessment. Full data for baseline and follow-up were available for 381 participants. From baseline to follow-up, there was no evidence for selective dropout (see [42, 43]). Previous mental disorders, previous deployment and previous experience of mission combat events at baseline did not predict participation at follow-up (see [42, 43]). Of the 381 participants for whom full data for baseline and follow-up were available, *n* = 23 were excluded from analysis, because they had not been deployed to Afghanistan as scheduled. The final sample, on which the analyses of the present study are based, thus comprised 358 participants. Of those 358 participants, 250 had reported at least one lifetime traumatic event at baseline. All analyses regarding PTS symptoms were therefore limited to this subsample of 250 participants.

### Procedures

The baseline assessment was conducted between 1 and 12 weeks before deployment at the home bases of the respective units. Baseline assessments took place between May 2011 and September 2011. Follow-up assessments were carried out about 12 months after return from deployment and took place from January 2012 to May 2013. Participants had spent an average of 5.3 months (*SD* = 1.46) on deployment. The 12-month time period between return from deployment and post assessment was chosen because the study aimed to investigate stress-related mental health conditions rather than short-term transient phenomena.

Participation in the study was strictly voluntary and confidential. Trained clinical psychologists completed informed consent procedures and conducted the assessments. All questionnaires were embedded in a computerized interview. Throughout the whole study procedure, pseudonymity of all participants was assured. The study was approved by the TUD ethics board (EK 72022010) and was performed according to ICH-GCP (Good Clinical Practice)-Guidelines.

### Measures

#### Perceived workplace social support

Perceived workplace social support provided by other military personnel was measured with an 11-item version of the *Deployment Social Support* of the Deployment Risk and Resilience Inventory (DRRI) designed to measure perceived social support with regard to fellow unit members, unit leaders and the military in general [44]. Sample items are “I could go to most people in my unit for help when I had a personal problem” and “My commanding officer(s) were interested in what I thought and how I felt about things”. Items were rated on a five-point scale (“Strongly disagree”, “somewhat disagree”, “neither agree nor disagree”, “somewhat agree”, “strongly disagree”). A total sum score was calculated from those items (theoretical range 11–55). At baseline (prior to deployment), the instruction of the questionnaire was to judge present unit support. At follow-up (after deployment), the instruction was to judge unit support during deployment as units did not remain the same after deployment. Internal consistency (averaged over baseline and follow-up) was α = 0.87.

#### Perceived general social support

Perceived general social support during the past 4 weeks (provided by family, friends, partner or significant other), measured at baseline and at follow-up, was assessed with four questions adapted from the *Social Network* Section of the National Comorbidity Survey-Replication [45]. Perceived social support by family and friends was assessed with the following two items: “How much can you rely on your family and friends for help if you have a serious problem – a lot (3), some (2), a little (1), or not at all (0); *not applicable*?” “How much can you open up to your family and friends if you need to talk about your worries – a lot (3), some (2), a little (1), or not at all (0); *not applicable*?” “Not applicable” was rated as missing.

Perceived social support by a partner or significant other was assessed with the following two items: “When you have a problem or worry, how often do you let your partner know about it – always (4), most of the time (3), sometimes (2), rarely (1), or never (0); *not applicable*?” “When you have a problem or worry, how often do you let another person close to you know about it – always (4), most of the time (3), sometimes (2), rarely (1), or never (0); *not applicable*?”. Of these two items, the one for which a participant had no missing value or reported the highest value was used to measure perceived social support by a partner or significant other. Items with a range of 0–4 were multiplied by 0.75 to be comparable to items that had a range of 0–3. Afterwards, the two items measuring perceived social support by family and friends and the item measuring perceived social support by a partner or significant other were summed up (theoretical range 0–9). Mean inter-item correlation (averaged over baseline and follow-up) was *r* = 0.25. Mean inter-item correlation is considered optimal when ranging between 0.20 and 0.40 [46].

#### Depressive and anxiety symptoms

Depressive and anxiety symptoms (past 7 days) were assessed with the Hospital Anxiety and Depression Scale German Version (HADS-D) [47] and were measured at baseline and at follow-up. The depression and the anxiety subscale of the HADS-D each consist of seven items that are rated on a four-point scale. The response scales are anchored differently for each item and measure either the severity of behavioral changes or the frequency or severity of symptoms. Sample items of the anxiety scale are “I get sudden feelings of panic” and “I get a sort of frightened feeling as if something awful is about to happen”. Sample items of the depression scale are “I still enjoy the things I used to enjoy” and “I can laugh and see the funny side of things”. A sum score was calculated for anxiety symptoms (theoretical range 0–21) and for depressive symptoms (theoretical range 0–21). Internal consistency, averaged over baseline and follow-up, was α = 0.71 for the depressive scale. For the anxiety scale, internal consistency was α = 0.75.

#### PTS symptoms

Posttraumatic stress (PTS) symptoms (past 4 weeks) were measured at baseline and at follow-up using the 17-item Posttraumatic Stress Disorder checklist (PCL) [48] in participants who reported at least one lifetime traumatic event at baseline (*N* = 250). In the present study, participants were instructed to answer the items with respect to the worst event. The PCL has been designed to assess the 17 symptoms of Posttraumatic Stress Disorder in DSM-IV [48]. Items are rated on a five-point scale (“not at all”, “a little bit”, “moderately”, “quite a bit”, “extremely”). Sample items are “Avoid thinking about or talking about the event or avoid having feelings related to it” and “Repeated, disturbing dreams of the event”. A total sum score was calculated from the items (theoretical range = 17–85). Averaged over baseline and follow-up, internal consistency was α = 0.89.

#### Neuroticism

Neuroticism (at baseline) was measured with the 2-item N*euroticism* scale from the Big-Five-Inventory-10 German Version [49]. Items are “I see myself as someone who is relaxed, handles stress well” and “I see myself as someone who gets nervous easily”. According to the guidelines, the mean value (theoretical range 1–5) was calculated from these two items if neither item was missing. Inter-item correlation was *r* = 0.32.

#### Number of previous traumatic events

Number of previous traumatic events (measured at baseline) represented the number of all traumatic events prior to deployment. A traumatic event was defined according to DSM-IV-TR A1 criterion [50]. Participants were provided with a list of traumatic events [51] which had been expanded to also include military-specific events.

### Statistical procedures

All analyses were performed with Stata 15.1 (StataCorp, 2017). For the depression, anxiety, PTS, perceived general social support and perceived workplace social support scores, individuals with more than 20% of items missing on the respective score were excluded from analysis. If answers were present for at least 80% of the items, missing values were replaced by individual means.

First, Spearman correlations (at baseline and at follow-up) of perceived general social support and perceived workplace social support with depressive, anxiety and PTS symptoms were calculated. Then, paired *t-*tests were performed to test whether average levels of perceived general social support and average levels of perceived workplace social support changed between baseline and follow-up measurement.

To examine the possible presence of subgroups of individuals with different perceived social support trajectories, a discrete mixture model for longitudinal data was applied using the Stata package “traj”, which is designed to identify distinctive clusters of individuals following similar trajectories over time [52, 53]. The procedure is based on a group modelling strategy that accommodates data groups with different parameter values for each group distribution [52, 53]. Maximum likelihood was used for the estimation of the model parameters. Since visual inspections revealed left skewed distributions for perceived workplace social support and perceived general social support, a censored normal model was applied, which is designed for the analysis of psychometric scales, that are (approximately) continuous and may be clustered at the minimum and/or the maximum of the scale [52, 53]. Several models were conducted to explore the number of subgroups that best fitted the data. The Aikake Information Criterion (AIC) and the Bayesian Information Criterion (BIC) were used to identify the model with the best fit for perceived general social support and for perceived workplace social support.

Afterwards, bidirectional longitudinal associations between perceived social support and psychological symptoms were examined. For the longitudinal analysis of bidirectional relationships, cross-lagged panel models are commonly applied [54]. In the basic example of a longitudinal cross-lagged panel model with only two measurement points, a cross-lagged panel model corresponds to a regression model in which the effect of a predictor at baseline (e.g. perceived general social support) on an outcome at follow-up (e.g. PTS symptoms) is estimated, controlling for the outcome (e.g. PTS symptoms) at baseline [54]. In line with this approach, several regressions models were calculated to examine bidirectional relationships between perceived social support and psychological symptoms. Graphical inspections revealed skewed distributions not only for the measures of perceived social support, but also for the scores of PTS, anxiety and depressive symptoms. All hypotheses were therefore tested using a generalized linear model assuming a gamma distribution, which accounts for skewed distributions. Associations were quantified with exponentiated regression coefficients (mean ratios, MRs). For the analysis of longitudinal associations, all scores were z-standardized to facilitate interpretability and comparability of coefficients. Since gamma regressions cannot be conducted with dependent variables containing negative values, scores being in the negative range after z-standardization were linearly transformed so that the smallest value of the score was zero.

Then, in a first step, longitudinal associations from perceived general social support before deployment and from perceived workplace social support before deployment to depressive symptoms, anxiety symptoms and PTS symptoms after deployment were calculated. As described above, all analyses were adjusted for the baseline value of the respective dependent variable (depressive symptoms, anxiety symptoms, or PTS symptoms). Second, longitudinal associations from depressive symptoms, anxiety symptoms and PTS symptoms before deployment to perceived general social support after deployment and to perceived workplace social support during deployment (measured retrospectively after deployment) were calculated. All analyses were, again, adjusted for the baseline value of the respective dependent variable (perceived general social support, or perceived workplace social support). Then, in an additional model, all analyses were further adjusted for neuroticism and number of previous traumatic events (both assessed before deployment) to investigate whether possible associations would be reduced when conditioning on neuroticism and previous traumatic events as potential common causes of perceived social support and psychological symptoms.

## Results

### Sample characteristics

Participants had a mean age of 27.2 years (*SD* = 6.3). There were 21.9% of participants who were married, 23.5% had children and 81.3% had a middle (10th grade) or high (equivalent to high school or higher) educational level. Mean length of service was 7.3 years (*SD* = 6.1). Mean values, standard deviations and pairwise correlations of all studied variables, at baseline and at follow-up, are shown in Table 1. As illustrated in Table 1, participants reported overall low levels of PTS, anxiety and depressive symptoms. At baseline, 5.7% of participants had a PTS severity score above the recommended cut-off for clinically relevant symptoms (score ≥ 30) [55]. At follow-up, 8.4% of participants demonstrated a PTS score ≥ 30. With respect to depressive symptoms, 4.0% participants at baseline and 5.6% of participants at follow-up were above the recommended cut-off for clinically relevant degrees of depression (score ≥ 8) [56]. Regarding anxiety symptoms, 8.5% of participants at baseline and 6.7% at follow-up had a score above the recommended cut-off point for clinically relevant anxiety (score ≥ 8).

**Table 1:** Means, standard deviations and Spearman correlations of studied variables (unstandardized) at baseline (BL) and at follow-up (FU)

						Percentiles					
Variable		*M*	*SD*	Min	Max	25th	75th	1	2	3	4
1. Perceived general social support	BL	7.8	1.1	2.3	9	7.3	8.3				
	FU	7.8	1.1	4.5	9	7.3	8.3				
2. Perceived workplace social support	BL	39.9	6.9	15	54	36	44	0.24**			
	FU	41.9	7.2	16	55	38	47	0.20**			
3. PTS symptoms	BL	19.8	5.2	17	52	17	20	−0.18*	− 0.23**		
	FU	20.6	6.7	17	59	17	20	−0.25**	− 0.09		
4. Anxiety symptoms	BL	3.3	2.7	0	14	1	5	−0.18**	− 0.17*	0.25**	
	FU	2.9	2.9	0	17	1	4	−0.34**	−0.21**	0.42**	
5. Depressive symptoms	BL	2.2	2.4	0	13	0	3	−0.22**	−0.33**	0.30**	0.54**
	FU	2.2	2.6	0	14	0	3	−0.35**	−0.30**	0.33**	0.55**

*M* and *SD* represent mean value and standard deviation, respectively. Min and Max represent minimum value and maximum value, respectively

*PTS* posttraumatic stress, *FU* Follow-up, *BL* Baseline

^*^p < .05

^**^p < .001

### Changes in perceived general social support and perceived workplace social support

Average levels of perceived general social support did not change between baseline (*M* = 7.81, *SD* = 1.11, Min = 2.25, Max = 9) and follow-up measurement (*M* = 7.78, *SD* = 1.10, Min = 4.5, Max = 9) (t(349) = − 0.63, *p* = 0.53). Descriptively, the average change in perceived general social support was *M* = − 0.03 (*SD* = 1.02).

Average levels of perceived workplace social support increased between baseline (*M* = 39.88, *SD* = 6.88, Min = 15, Max = 54) and time of deployment (retrospectively measured at follow-up) (*M* = 41.92, *SD* = 7.15, Min = 16, Max = 55) (t(344) = 5.51, *p* < .001). Descriptively, the average change in perceived workplace social support was *M* = 2.11 (*SD* = 7.12).

### Trajectories of perceived general social support and perceived workplace social support

Regarding perceived general social support, results from the discrete mixture models indicated that the model that fitted the data best was the one assuming only one trajectory. Also for perceived workplace social support, the one trajectory-solution was the best fit to the data. Thus, there were no distinguishable subgroups of trajectories of perceived workplace social support or of trajectories of perceived general social support. All tested models and the corresponding information criteria are shown in the supplemental material (Table S1, Table S2).

### Associations between perceived social support at baseline and psychological symptoms at follow-up

All associations between perceived social support at baseline and psychological symptoms at follow-up are shown in Table 2.

**Table 2:** Associations of perceived social support (general and workplace) at baseline with psychological symptoms (anxiety, depressive and PTS symptoms) at follow-up

Variable	Depressive symptoms			Anxiety symptoms			PTS symptoms^a^		
	*MR*	*p*	95% CI	*MR*	*p*	95% CI	*MR*	*p*	95% CI
**Perceived general social support**	0.84	.005	[0.74, 0.95]	0.91	.065	[0.82, 1.01]	1.13	.361	[0.87, 1.49]
Adjusted model	0.84	.007	[0.74, 0.95]	0.94	.274	[0.85, 1.05]	1.20	.165	[0.93, 1.55]
**Perceived workplace social support**	0.82	.001	[0.72, 0.92]	0.90	.063	[0.80, 1.01]	1.11	.486	[0.83, 1.47]
Adjusted model	0.82	.001	[0.72, 0.93]	0.94	.256	[0.84, 1.05]	1.05	.703	[0.80, 1.38]

Adjusted Model: adjusted for neuroticism and number of previous traumatic events at baseline

*PTS* posttraumatic stress, *MR* Mean ratio. All variables are z-standardized

^a^With respect to PTS symptoms the analysis sample was limited to *N* = 250 participants who had reported a lifetime traumatic event at baseline

Higher perceived general social support at baseline (*MR* = 0.84, 95% CI = [0.74, 0.95], *p* = .005) and higher perceived workplace social support at baseline (*MR* = 0.82, 95% CI = [0.72, 0.92], *p* = .001) were associated with lower depressive symptoms at follow-up. These associations were still present when neuroticism and number of previous traumatic events were added to the model (general: *MR* = 0.84, 95% CI = [0.74, 0.95] *p =* .007; workplace: *MR* = 0.82, 95% CI = [0.72, 0.93] *p =* .001). There was no evidence that coefficients of the unadjusted model and the adjusted model differed (general: χ^2^(1) = 0.02, *p* = .901; workplace: χ^2^(1) = 0.02, *p* = .883).

There was no evidence for associations between perceived general social support at baseline (*MR* = 0.91, 95% CI = [0.82, 1.01], *p* = .065) or perceived workplace social support at baseline (*MR* = 0.90, 95% CI = [0.80, 1.01], *p* = .063) and anxiety symptoms at follow-up. However, *p*-values were close to the significance threshold. When adjusted for neuroticism and previous traumatic events, the point estimates were closer to 1 and *p*-values were clearly above the significance threshold *(*general: *MR* = 0.94, 95% CI = [0.85, 1.05], *p* = .274; workplace: *MR* = 0.94, 95% CI = [0.84, 1.05], *p* = .256). Coefficients of the unadjusted model and the adjusted model were significantly different (general: χ^2^(1) = 5.76, *p* = .016, workplace: χ^2^(1) = 4.63, *p* = .031).

Similar to anxiety symptoms, there was also no association between perceived general social support at baseline (*MR* = 1.13, 95% CI = [0.87, 1.49], *p* = .361) or perceived workplace social support at baseline (*MR* = 1.11, 95% CI = [0.83, 1.47], *p* = .486) and PTS symptoms at follow-up.

### Associations between psychological symptoms at baseline and perceived social support at follow-up

All associations between psychological symptoms at baseline and perceived social support at follow-up are shown in Table 3.

**Table 3:** Associations of psychological symptoms (anxiety, depressive and PTS symptoms) at baseline with perceived social support (general and workplace) at follow-up

Variable	Perceived general social support			Perceived workplace social support		
	*MR*	*p*	95% CI	*MR*	*p*	95% CI
**Depressive Symptoms**	0.96	.024	[0.93, 0.99]	0.98	.253	[0.96, 1.01]
Adjusted model	0.97	.088	[0.93, 1.005]	0.98	.192	[0.95, 1.01]
**Anxiety Symptoms**	0.98	.165	[0.94, 1.01]	0.99	.452	[0.96, 1.02]
Adjusted model	0.98	.410	[0.95, 1.02]	0.99	.324	[0.96, 1.01]
**PTS symptoms**^**a**^	0.95	.017	[0.91, 0.99]	1.01	.492	[0.98, 1.05]
Adjusted model	0.95	.019	[0.91, 0.99]	1.01	.657	[0.97, 1.05]

Adjusted Model: adjusted for neuroticism and number of previous traumatic events at baseline

*PTS* posttraumatic stress, *MR* Mean ratio. All variables are z-standardized

^a^With respect to PTS symptoms the analysis sample was limited to *N* = 250 participants who had reported a lifetime traumatic event at baseline

Higher depressive symptoms at baseline were associated with lower perceived general social support at follow-up (*MR* = 0.96, 95% CI = [0.93, 0.99], *p* = .024). The association was almost similar when also adjusted for neuroticism and previous traumatic events, only the confidence interval was somewhat broader with a *p*-value exceeding the 5% threshold (*MR* = 0.97, 95% CI = [0.93, 1.005] *p =* .088). There was no evidence that coefficients of the unadjusted model and the adjusted model differed (χ^2^(1) = 1.08, *p* = .299). There was no association between depressive symptoms at baseline and perceived workplace social support during deployment (retrospectively measured at follow-up) (*MR* = 0.98, 95% CI = [0.96, 1.01], *p* = .253). To be noted, the found association between depressive symptoms and baseline and perceived general social support at follow-up was smaller than the reverse association between perceived general social support at baseline and depressive symptoms at follow-up (χ^2^(1) = 5.24, *p* = .022).

There was no association between anxiety symptoms at baseline and perceived general (*MR* = 0.98, 95% CI = [0.94, 1.01], *p* = .165) or workplace (*MR* = 0.99, 95% CI = [0.96, 1.02], *p* = .452) social support at follow-up.

Similar to depressive symptoms, higher PTS symptoms at baseline predicted lower perceived general social support at follow-up (*MR* = 0.95, 95% CI = [0.91, 0.99], *p* = .017). This association remained similar when also adjusted for neuroticism and previous traumatic events (*MR* = 0.95, 95% CI = [0.91, 0.99], *p =* .019). There was no evidence that coefficients of the unadjusted model and the adjusted model differed (χ^2^(1) = 0.05, *p* = .815). There was no association between PTS symptoms at baseline and perceived workplace social support during deployment (measured at follow-up) (MR = 1.01, 95% CI = [0.98, 1.05], *p* = .492).

## Discussion

The aim of the present study was to investigate the stability of perceived social support as well as bidirectional associations between perceived social support and psychological symptoms in the context of stressful event exposure. The study was conducted in a sample of German soldiers assessed before and after deployment to Afghanistan. Average levels of perceived general social support did not change over the deployment period, while average levels of perceived workplace social support slightly increased. The latter is plausible, since a joint foreign mission in which members of a unit spend a considerable amount of time together, face stress together and have to accomplish a common task, can strengthen group cohesion [57, 58]. However, the reported average increase in perceived workplace social support was rather small. Taken together, the results of this study provide no conclusive evidence that average levels of perceived social support decline or increase markedly in the context of stressful event exposure. Regarding intra-individual stability, there was no evidence for subgroups of individuals with distinguishable trajectories of perceived social support. Therefore, perceived social support seems to be relatively stable for the majority of individuals. These findings are in line with other studies that conceptualize perceived social support as a rather stable variable [16, 17]. It should be kept in mind that in a high-risk occupational context such as the military, stressful event exposure is expected and prepared for [22]. Moreover, soldiers are collectively exposed to similar environmental stressors with support and responsibility for each other being important parts of military culture and training [22]. This could make it easier for soldiers to maintain perceptions of well-functioning social ties even through stressful event exposure. Similar considerations might apply to other contexts in which individuals are collectively exposed to stressful events they have been prepared for, such as firefighting, law enforcement or first aid. In the present study, there was approximately one and a half years between baseline and follow-up measurement. Perceived social support might be less stable if individuals are exposed to an increasing number of stressful events over a longer period of time [22].

The main aim of this paper was to investigate bidirectional associations of perceived social support (workplace and general) with psychological symptoms (anxiety, depression and PTS symptoms) in the context of stressful event exposure.

The association between higher perceived social support and lower depressive symptoms is in line with previous studies [59–61]. However, to our best knowledge, this is the first study to investigate the association between perceived social support and depressive symptoms when measured before and after stressful event exposure. With respect to depressive symptoms, results of the present study are in line with a stress-buffering model, which suggests that higher perceived social support can protect against the adverse effects of stressful event exposure by redefining the harms and demands of the situation [15]. The perception that others can and will provide helpful resources could increase a person’s perceived ability to cope with a stressful situation [15]. We did not find evidence for an association between perceived social support and PTS symptoms. This is in line with several previous longitudinal studies that investigated bidirectional associations and also found an association from higher PTS symptoms to lower perceived social support, but no [32–34] or only minor [39] evidence for the reverse association. However, there is also a considerable number of studies that demonstrated a longitudinal association between higher perceived social support and lower PTS symptoms [14, 25]. Consistent with these equivocal findings, meta-analytic analysis has shown that study results on the association between perceived social support and PTS symptoms are highly heterogeneous [14]. In line with this, the stress-buffering and matching hypothesis [15, 62] suggests that, in the context of stressful event exposure, perceived social support is only beneficial in cases where there is a close match between the needs and coping requirements elicited by the stressful event and the kind of social support that is perceived as available. It has been suggested that certain types of stressful events (e.g. loss of physical capacity) typically elicit certain needs and therefore are particularly responsive to certain forms of social support (e.g. tangible support) [39, 62]. However, it has to be noted that needs elicited by stressful event exposure might vary greatly among individuals having been exposed to the same stressful event. Individuals might differ in whether they experience an event as particularly threatening to, for instance, their need for attachment and belonging, self-esteem, or self-efficacy. Moreover, individuals might not only differ with respect to needs being particularly salient, but also in the extent to which they are able to accept and benefit from provided social support. It has been suggested that social support is only beneficial if it is perceived by an individual as being reciprocal [63]. In summary, it is likely that certain conditions must be met to be able to benefit from perceived social support. It could be that this is particularly pronounced for trauma-related symptomatology [64]. In line with this, in the present study, there was an association between perceived social support and depressive symptoms, but not between perceived social support and PTS symptoms. One might speculate that a protection from PTS symptomatology requires a more specific match between current needs (e.g. strengthening of self-esteem, strengthening of self-efficacy, belonging to a group, having a close relationship) and types of perceived social support, whereas (subclinical) depressive symptoms could be more responsive to general perceptions of social support. Moreover, as lifetime prevalence is higher for depressive disorders than for Posttraumatic Stress Disorder [65], more people might relate to and be able to understand depressive symptoms, and to offer corresponding support.

Regarding the reverse relationship, both higher depressive as well as higher PTS symptoms contributed to lower perceived social support in the present study. With respect to depressive symptoms, this pattern could be indicative of a vicious circle in which individuals with higher depressive symptoms have lower perceived social support and in which lower perceived social support then contributes to further worsening of depressive symptomatology. Different theories offer explanations why higher depressive and higher PTS symptoms might lead to lower perceived social support. One possibility could be that impaired social abilities associated with both depressive and PTS symptoms contribute to a decrease in perceived social support. Problems regarding social functioning could thereby manifest on affective, cognitive and behavioral levels [66]. Among others, depressive and PTS symptoms are associated with problems in social cognition, that is the identification, perception and interpretation of socially relevant information [28, 67]. Moreover, individuals with depressive [27, 68, 69] and PTS symptoms [69–72] report more distressing social emotions and associated maladaptive social cognitions. These feelings and cognitions relate, for example, to shame, guilt, social alienation and revenge [68–72]. On a behavioral level, individuals with depressive and PTS symptoms report problems regarding emotional self-disclosure [73, 74] and show decreased reward-oriented social behavior [75, 76]. To conclude, impaired social abilities might hinder individuals with elevated depressive and PTS symptoms to access social support after stressful event exposure. Regarding the reverse relationship, perceived social support could protect against depressive symptoms via a stress-buffering mechanism, whereas protection against PTS symptomatology might require a more specific match between individual needs and the type of perceived social support.

This study has several limitations. (1) The findings described above relate to measures of perceived social support. We have no information on the extent to which levels of perceived social support reflect the amount of objective social support. However, it has previously been shown that measures of social support that are more objective, often captured by the concepts of enacted or received social support, only explain a small proportion of the variance in perceived social support [77]. Moreover, somewhat objective measures of social support, including enacted social support or network size, are less strongly related to mental health than perceived social support [14]. Therefore, it is considered unlikely that differences in perceived social support and in psychological symptoms resulted largely from differences in objective social support. To bring further clarity to this issue, a valuable goal for future studies would be to examine changes in objective social support (e.g., as a result of the loss of friends and family, separation from a partner, or other crises) in relation to changes in perceived social support and psychological symptoms. (2) Participants reported overall low levels of depressive, anxiety and PTS symptoms. Thus, only assumptions about subclinical symptom levels can be made. Although the results should therefore be interpreted with caution with regard to clinical relevance, findings on subclinical symptoms are valuable for the development of early preventive measures. (3) The found associations were rather small. This could in part be due to ceiling effects in reported perceived social support and bottom effects in reported psychological symptoms. These limited variances could have led to a potential underestimation of associations. (4) All analyses regarding PTS symptoms were limited to the subsample of *N* = 250 participants, who had reported a lifetime traumatic event at baseline. As associations regarding depressive and anxiety symptoms were examined in the larger total sample of *N* = 358 participants, it is possible that differences in statistically significant associations might be the result of differences in statistical power. However, this risk is deemed unlikely given a comparable width of confidence intervals, and effect sizes close to zero for associations in the trauma exposed sample that were below the significance threshold. (5) At follow-up (after deployment), perceived workplace social support was assessed with respect to time during deployment as units did not remain the same after deployment. Information provided on perceived workplace social support during deployment might therefore have been subject to memory bias. (6) Stressful military events as investigated in this study are primarily characterized by occupational high-risk demands [22]. Further research is needed to determine whether the associations found in this study also apply to other types of stressful contexts. Moreover, although military populations are well suited to investigate social support in the context of exposure to stressful events, the specific characteristics of a male military sample limit the generalizability of the findings. (7) German translations were not available for several scales and items had to be translated for study purposes. Therefore, the psychometric quality of these questionnaires cannot be evaluated conclusively. (8) At follow-up, soldiers were investigated about 12 months after return from deployment. In the time between return and follow-up assessment, there may have been impacts on psychological symptoms and on perceived social support that were not assessed as part of the study. However, these potential influences occurred after the predictor and therefore cannot be a confounding factor in the potential causal association between predictor and outcome [78].

Despite these limitations, several important implications can be drawn from the results of this study. First, the results suggest that perceived social support is not strongly influenced by environmental stressors, at least in the medium term. This would imply that interventions aimed at promoting perceived social support can be of lasting benefit to individuals and could increase their resilience [79]. If efforts to strengthen perceived workplace support and perceived general social support are successful prior to a stressful event (e.g., military deployment), higher perceived social support could function as a potential protective factor both during and after the stressful event. Results of the present study further suggest that higher perceived general social support and higher perceived workplace social support before stressful event exposure might be protective against depressive symptoms after stressful event exposure. Our results further support the notion that already subclinical levels of PTS and depressive symptoms before stressful event exposure might contribute to lower perceived general social support after stressful event exposure. These relationships suggest that it is important to monitor and regularly screen individuals who are exposed to severe stressful events. This would include screening not only for full blown mental disorders, but also for subclinical symptom manifestations, as these could promote a problematic cycle of higher symptoms and lower perceived social support. Indicated prevention might be offered to individuals with subclinical levels of, for instance, depressive or PTS symptoms. An important aspect of such preventions could be the training of social abilities. Recent developments suggest that social abilities, including social perception, mentalizing, empathizing and prosocial motivation, are malleable and can be learned and unlearned [66, 80]. This is especially true for individuals who do not (yet) have a chronic or severe mental disorder, which may be associated with more rigid and inflexible social behavior, emotions and cognitions [66]. For individuals with subclinical depressive or PTS symptoms, trainings addressing social abilities might also include coping with distressing social emotions and associated maladaptive social cognitions (e.g. related to shame, revenge, social alienation). Such trainings could both help to prevent the development of full-blown mental disorders and improve perceived social support.

Future studies should further explore bidirectional associations between perceived social support and different psychological symptoms over the course of stressful event exposure, with a particular focus on conditions that enable individuals to benefit from social support. Such studies should ideally cover a longer period of time and include several measurement points. It may be helpful to capture the extent to which individuals have perceived social support that meets the basic needs of self-esteem, attachment, orientation and control, and pleasure [81]. A valuable target for future studies would be to identify subgroups of individuals (depending on, for instance, symptomatology and number of experienced traumatic events) that benefit differently from different forms of perceived social support. If the results of the present study are confirmed in future studies, studies should look into mechanisms that could lead from higher perceived social support to lower depressive symptoms and from higher PTS and higher depressive symptoms to lower perceived social support. With respect to the latter association, distressing social emotions and associated maladaptive social cognitions could play an important mediating role. It would be a valuable goal for future studies to investigate whether trainings that address socio-cognitive and socio-affective abilities, including coping with distressing social emotions, are helpful in increasing perceived social support and in preventing the development of full-blown mental disorders.

## Conclusions

Our study extends previous findings on the interplay between psychological symptoms and perceived social support in the context of stressful event exposure by investigating perceived social support and psychological symptoms before and after military deployment as a form of stressful event. Our results indicate that individuals with heightened depressive or PTS symptoms before exposure to a stressful event have poorer abilities or opportunities to access general social support after stressful event exposure. Our results further suggest that higher perceived workplace and general social support prior to a stressful event could protect against post-event depressive symptoms. Perceived social support, however, may need to be more tailored to individual needs to provide protection against PTS symptoms. In our study, perceived social support remained relatively stable under exposure to an environmental stressor. Interventions that focus on strengthening perceived general or workplace social support before a stressful event might therefore have benefits that persist during and after stressful event exposure. Interventions focusing on the training of social abilities, with particular attention to distressing social emotions and associated maladaptive social cognitions, might be helpful in enhancing perceived social support, especially for individuals with PTS or depressive symptoms. Particularly with regard to PTS symptomatology, identifying subgroups of individuals who benefit differently from perceived social support could be a valuable target for future studies.

## Supplementary Information

**Additional file 1: Table S1.** Results of group-based trajectory modelling for perceived workplace social support. **Table S2.** Results of group-based trajectory modelling for perceived general social support.

## Data Availability

The datasets generated and analyzed during the current study are not publicly available due to privacy restrictions (i.e., pseudonymized data collection and no informed consent about public availability of the raw data from the participants during data collection in 2011–2012) but are available from the corresponding author (Sebastian Trautmann) on reasonable request.

## Abbreviations

PTS: Posttraumatic Stress
MR: Mean ratio
