# Supplementary material for: Examining bidirectional associations between perceived social support and psychological symptoms in the context of stressful event exposure: a prospective, longitudinal study
Source: BMC Psychiatry. 2022 Nov 28;22:736. doi: 10.1186/s12888-022-04386-0 (PMC9703701; doi:10.1186/s12888-022-04386-0)
Supplement: Supplementary file 1 — Additional file 1: Table S1. Results of group-based trajectory modelling for perceived workplace social support. Table S2. Results of group-based trajectory modelling for perceived general social support. [file 12888_2022_4386_MOESM1_ESM.docx]

**Table S1**

*Results of group-based trajectory modelling for perceived workplace social support*

| Model | AIC | BIC |
| --- | --- | --- |
| Model 1: one trajectory | -2359.75 | -2366.59 |
| Model 2: two trajectories | -2323.83 | -2337.49 |
| Model 3: three trajectories | -2315.53 | -2336.02 |
| Model 4: four trajectories | -2318.53 | -2345.85 |
| Model 5: five trajectories | -2303.61 | -2337.76 |
| Model 6: six trajectories | -2306.61 | -2347.59 |

**Table S2**

*Results of group-based trajectory modelling for perceived general social support*

| Model | AIC | BIC |
| --- | --- | --- |
| Model 1: one trajectory | -1108.85 | -1115.70 |
| Model 2: two trajectories | -1070.17 | -1083.85 |
| Model 3: three trajectories | -1039.35 | -1059.88 |
| Model 4: four trajectories | -1024.11 | -1051.49 |
| Model 5: five trajectories | -1023.47 | -1057.69 |
| Model 6: six trajectories | -1024.24 | -1065.30 |
